# Supplementary material for: Acceptance and compliance with micronutrient powder (MNP) among children aged 6–23 months in northern Nigeria
Source: PLOS Glob Public Health. 2022 Oct 17;2(10):e0000961. doi: 10.1371/journal.pgph.0000961 (PMC10022258; doi:10.1371/journal.pgph.0000961)
Supplement: S1 Dataset — (ZIP) [file pgph.0000961.s008.zip › S8_Dataset/Direct Observations.zip!/Direct Observations/06072015_A_GA_AD_2_DO.pdf]

1.1.1 Instrument F –Direct Observation Form

**<<Instrument F Direct Observation Form>>**<br>**PI Name:** PragyaMathema,Stephen Kodish<br>**Study Title:** Formative research to develop an infant and young child feeding (IYCF) intervention with micronutrient powder (MNP) for children aged 6 – 23 months<br>**PI Version/Date:** Version1.0/January 24, 2015

Date: _6/07/2015______________Location: __DADIRI CMMUNITY, GANYE LGA________

Data Collector: __AISHA ALIYU DAHIRU____Participant ID: ____Mud House with tree in front______

Main goals of this direct observation are to answer the following questions:<br><br>1. In this household, what would be the best way(s) to give nutritional supplements to young children? Why?<br><br>2. In this household, describe the food sharing that you observe throughout the meal. Are there any predictable patterns?<br><br>3. In this household, who is favored and how is this favoritism expressed during meal preparation and eating?

(TEAR OFF AT PERFORATED DOTTED LINE BELOW)

PARTICIPANT ID: _________

Household Composition Table (Fill out as you do data collection)

INITIALS	SEX (M/F)	AGE (estimated)	Relationship to household	Description
M	F	2years	Mother	Wearing red wrapper with black shirt
B	M	18 months	Baby boy	Wearing green shirt and trouser
H	M	35years	Head of household	Wearing white kaffen and is dark
NB	F	15months	Neighbors child	Wearing pink skirt with red shirt
N1	F	29years	Neighbor	Wearing brown gown wrapper with a head gear

N2	F	27years	Neighbor	Wearing green wrapper with a white head gear
B2	M	8years	Older son	Wearing brown kaffen
B3	M	6years	Older son	Wearing green shirts with yellow shirt

PARTICIPANT ID: _________

TIME	ACTOR INITIALS	RECIPIENT INITIALS	WHAT HAPPENED	FOOD	Reactivity
11:00	M	B	Carrying child on her back		
11:05	M		Sweeping		
11:10	M		Sweeping		
11:15	M		Sweeping		
11:20	M		Washing hands with soap and water		
11:25	M		Serving kunu into cup from flask	Kunu dawa	
11:30	M	B	Feeding child the kunu while sitting on her lap	Kunu dawa	
11:31	M	B	Feeding child kunu. He is taking the kunu with enthusiasm	Kunun dawa	
11:32	M	B	Feeding the child kunu the child across and uncross his legs	Kunun dawa	

TIME	ACTOR INITIALS	RECIPIENT INITIALS	WHAT HAPPENED	FOOD	Reactivity
			clapping hands		
11:33	M	B	Feeding child kunu	Kunun dawa	
11:34	M	B	Still feeding the child kunu and he is willing to drink the kunu	Kunun dawa	
11:35	M	B	feeding the child the last spoon full of kunu	Kunun dawa	
11:40	M	B	Carrying child on her laps		
11:45	H		Chatting with mother		
11:50	H	B	Playing with child around the house		
11:55	H	B	Playing with child		
12:00	H	B	Plaiting neighbors child		
12:05	B		Standing beside the father		
12:10	B		Playing beside the father on the ground the father is sitting on a stool under a tree		
12:10	M	NB	Still plaiting neighbors child		
12:15	H	B	Sitting on fathers laps carrying child on his laps. Playing with the child.		
12:20	M	N1	Chatting with neighbor		
12:20	B		Playing with father’s mobile phone		
12:25	M	NB	Still plaiting neighbors child		
12:30	M	NY	Chatting with the child		
12:30	H	B	Chatting with the child		
12:35	H	B	Showing child something on the phone when is making the child to laugh		
12:40	H	B	Carrying child on his laps playing with him		
12:45	M	N1	Chatting with neighbor		

TIME	ACTOR INITIALS	RECIPIENT INITIALS	WHAT HAPPENED	FOOD	Reactivity
12:50	M	NB	Finishing up plaiting the neighbors child		
12:50	H	B	Giving the child water to drink from a large silver water cup		
12:55	M		Washing hands with soap and water after plaiting with him		
13:00	H	B	Carrying child on his laps playing with him		
13:05	M		Sitting under the lime tree		
13:10	H		Performing ablution to go to mosque		
13:05	B		Crying, he wants to follow father to the mosque		
13:15	M	B	Carrying child on her laps consoling him		
13:20	M	B	Breast feeding the child	Breast milk	
13:25	M	B	Breast feeding the child	Breast milk	
13:30	M	M, B	Breast feeding the child	Breast milk	
13:35	H	B	Chatting with the mother and child		
13:40	M	B	Carrying child on her laps		
13:45	M	B	Playing with child, lifting him up and down. The child is laughing		
13:50	B		Crying because mother kept him on the mat and stood up		

TIME	ACTOR INITIALS	RECIPIENT INITIALS	WHAT HAPPENED	FOOD	Reactivity
13:50	M	B	Carrying child and going into the room		
13:55	M		Mother playing		
13:55	B		Playing beside the mother while she is praying		
14:00	M	B	Carrying child on her laps		
14:05	M	B	Playing with child on her laps		
14:10	B		Playing on a mat on the veranda alone		
14:15	B	NB	Playing with neighbors child on the mat under the veranda		
14:20	B	NB	Playing with the neighbors child on the mat		
14:25	M		Sitting under lime tree on a small stool		
14:30	M	N2	Chatting with neighbors		
14:35	M	N1, N2	Chatting with neighbors		
14:40	B	NB	Playing with neighbors child		
14:45	M		Picking dirt out of mane she brought out from the room		
14:50	M	N1, N2	Chatting with neighbors while picking dirt out of maize. They are assisting her		
14:55	B		Playing alone on the mat on the veranda		
15:00	B2	B	Joins child on the mat playing with him		
15:05	B2	B	Playing with child under the veranda		
15:10	B		Playing around the veranda with the older son		

TIME	ACTOR INITIALS	RECIPIENT INITIALS	WHAT HAPPENED	FOOD	Reactivity
15:15	B3		Washing hands with water		
15:20	M	N1,N2	Chatting with neighbors while picking dirt out of maize		
15:25	B2	B	Carrying child on his back moving around the house		
15:25	B		Laughing and screaming on older son's back		
15:30	B2	B, M	Handing the mother the child		
15:35	M	B	Carrying child on her laps		
15:40	B	NB	Standing beside mother playing with neighbors child		
15:45	M	B	Carrying child on her laps breast feeding him	Breast milk	
15:50	M	B	Breast feeding the child	Breast milk	
15:55	M	B	Breast feeding the child	Breast milk	
16:00	M	B	Breast feeding the child	Breast milk	
16:05	B2	B	Carrying child from mother's laps		
16:10	B2	B	Carrying child		
16:15	M	B	Carrying child on her laps		
16:20	B		Grabbing mothers breast to breast feed		
16:20	M	B	Watching as the child tries to breast feed	Breast milk	
16:25	M	B	Breast feeding the child	Breast milk	
16:30	M	B	Turning the child to breast feed from the other breast	Breast milk	
16:35	M		Starting fire		
16:40	M		Starting fire		

16:45	M		Waiting for water to boil		
16:45	M		Mixing groundnut paste in a bowl with water	Groundnut paste	
16:50	M		Adding guinea corn flouring into groundnut paste mixture	Groundnut paste	
16:55	M		Adding mixture into boiling water on fire		
17:00	M		Stirring mixture to make kunun gyeda	Kunun gyeda	
17:05	M		Kunun gyeda is ready to be served		
TIME	ACTOR INITIALS	RECIPIENT INITIALS	WHAT HAPPENED	FOOD	Reactivity
17:05	M		Serving kunu into flask	Kunun gyeda	
17:05	M		serving a portion of kunu into a small cup	Kunun gyeda	
17:10	M		Serving kunu to cool before feeding child	Kunun gyeda	
17:15	M		Adding MNP into the warn kunu	Kunun gyeda	
17:15	M		Stirring MNP to ensure it dissolves into the kunu and covers it		
17:20	M		Checking the firewood		
17:20	M		Putting another pot of water on fire		
17:25	M	B	Feeding the child the kunu that was mixed with MNP. The child is taking the kunu like he previously with enthusiasm	Kunun gyeda	
17:26	M	B	Feeding the child kunu mixed with<br>MNP and the child was drinking the kunu with a spoon sitting on the mother’s lap	Kunun gyeda	
17:27	M	B	Still feeding the child	Kunun gyeda	
17:28	M	B	Still feeding the child	Kunun gyeda	
17:29	M	B	Feeding the child kunu	Kunun gyeda	
TIME	ACTOR INITIALS	RECIPIENT INITIALS	WHAT HAPPENED	FOOD	Reactivity

17:30	M	B	Washing child’s hands and mouth after feeding		
17:30	M		Checking the firewood		
17:35	M		Waiting for water to boil water to add guinea corn flour which is kept on the side	water guinea corn flour	
17:40	M		Adding guinea corn flour into the boiling water on the fire	water guinea corn flour	
17:40	B		Playing around the house, running around		
17:45	M		Meal is ready to be served	Guinea corn meal	
17:45	M		Serving meal into a single bowl	Guinea corn meal	
17:50	M		Washing hands		
17:50	M		Grinding onion and pepper	Onion pepper	
17:55	M		Adding ground pepper and onion into pot on fire	Onion pepper	
18:00	B		Running around the house laughing		
TIME	ACTOR INITIALS	RECIPIENT INITIALS	WHAT HAPPENED	FOOD	Reactivity
18:05	M		Adding bulbar flour into the boiling pot and stirring	Baobab flour	
18:10	M		Soup is ready to be served	Baobab flour (miyan kuka)	
18:10	M		Serving soup into a single bowl	Miyan kuka	
18:15	M		Washing utensils used in cooking		
18:20	M		Washing utensils used in cooking		
18:25	B		Playing on the mat undr the veranda		
18:35	M		Sweeping the cooking area		
18:40	M		Sweeping		
18:45	M		Washing hands with soap and water		

18:50	M		Serving meal to the husband and children in separate bowls. The husband alone and the children eat together	Tuwon dawa	
TIME	ACTOR INITIALS	RECIPIENT INITIALS	WHAT HAPPENED	FOOD	Reactivity
18:51	M		Serving food for herself in a separate bowl	Tuwon dawa, miyan kuka	
18:52	M	B	Eating together with the child. Feeding the child from her bowl	Tuwon dawa, miyan kuka	
18:53	H		Eating alone in a separate bowl	Tuwon dawa, miyan kuka	
18:54	M	B	Assisting the child to eat from her bowl	Tuwon dawa, miyan kuka	
18:55	B2		Eating together with brother from the same bowl	Tuwon dawa, miyan kuka	
18:56	B3		Eating together with older son from the same bowl	Tuwon dawa, miyan kuka	
18:57	M	B	Still assisting child to eat from her bowl	Tuwon dawa, miyan kuka	
18:57	H		Eating alone from a separate bowl	Tuwon dawa, miyan kuka	
18:59	M	B	Washing the child's hands and mouth		
19:00	M	B	Giving child water to drink from a large silver cup	Water	

TIME	ACTOR INITIALS	RECIPIENT INITIALS	WHAT HAPPENED	FOOD	Reactivity
19:05	M	B	Carrying child on her back		
19:05	H		Washing hands after eating		
19:05	B2		Washing hands supervised by the father		
19:05	B3		Washing hands supervised by the father		
19:10	B		Sleeping on mother's back		
19:10	B2		Drinking water from the large silver cup	Water	
19:10	B2		Drinking water from the large silver cup	Water	
19:15	M		Lying child down on the bed for the night		
19:20	H	M, B2, B3	Chatting with mother and older son under the veranda		
19:25	H	M, B2, B3	Chatting with mother and children		
19:25	B		Sleeping in the room		
19:30	B		Sleeping		

TIME	ACTOR INITIALS	RECIPIENT INITIALS	WHAT HAPPENED	FOOD	Reactivity

TIME	ACTOR INITIALS	RECIPIENT INITIALS	WHAT HAPPENED	FOOD	Reactivity

TIME	ACTOR INITIALS	RECIPIENT INITIALS	WHAT HAPPENED	FOOD	Reactivity

PARTICIPANT ID: _________

Primary Questions (Complete after you complete data collection):

From what you have seen observing THIS HOUSEHOLD, please answer the following questions in detail:

Question	Write your detailed response here
1. In this household, what would be the best way(s) to give nutritional supplements such as an MNP to young children to ensure compliance?<br><br>Why?	The best way to ensure compliance is to keep reminding the mother of the importance of the MNP to her child.<br><br>The mother should ensure she has a separate bowl for feeding the child MNP with.
2. In this household, who is favored during meal preparation or feeding?<br><br>How is this favoritism expressed?	The parents and older children are favoured because the last meal was solid.<br><br>The father and the older children were favoured because the quantity of their food was more than the mother's.<br><br>The little child was feed carefully by the mother
3. How could we promote the MNP supplement for this family that would attract their attention?<br><br>Who would they listen to?	MNP can be promoted to this family through the mother.<br><br>The father can also be an influence because he was asking if the child was given the MNP when he came into the house.<br><br>A radio was playing at the neighbors and it was heard in his household even though they did not put theirs on.

4. In this household, what would be the best ways to promote the MNP but limit how much it would be shared?<br><br>Why?	
5. What other issues and concerns did you see with this family that are important for us to consider with regard to introduction of an MNP?<br><br>Why?	
6. Based on your experience observing this child today, do you believe reactivity negatively impacted your ability to see the food-related behaviors of the child?<br><br>Please explain your answer in detail.	
